# Targeting vision-threatening disease in EyeArt-based diabetic retinopathy screening: A letter in response to: “Evaluation of the degree of agreement in the diagnosis of diabetic retinopathy between ophthalmologists and EyeArt”

**DOI:** 10.1186/s40942-025-00787-x

**Published:** 2026-01-27

**Authors:** Henry Bair

**Affiliations:** Wills Eye Hospital, 840 Walnut St, Philadelphia, PA 19107 USA

## Abstract

Guedes et al. demonstrate excellent agreement between EyeArt and ophthalmologist grading for any diabetic retinopathy (DR) within a regional screening program, with near-perfect binocular sensitivity and high specificity. Building on these findings, this letter argues that screening programs are primarily justified by the prevention of vision-threatening DR (vtDR), and that performance metrics should therefore prioritize vtDR rather than any DR as the primary endpoint. The letter highlights how the current operating point, optimized for any DR, may not reflect an optimal balance between sensitivity, specificity, and downstream referral burden in real-world workflows. It also discusses how the study’s OCT-based risk factors support the development of multimodal and risk-integrated AI systems that combine fundus imaging, OCT, and clinical variables to triage vtDR more efficiently and personalize screening intervals.

## To the Editor

Guedes et al. report excellent agreement between EyeArt and ophthalmologist grading for diabetic retinopathy (DR) in a regional screening program, with binocular sensitivity of 100% and specificity of 93.5% [1]. This strengthens the case for autonomous AI in DR screening. However, an aspect that warrants further exploration is the choice of “any DR” as the primary endpoint rather than vision‑threatening DR (vtDR).

From a health‑system perspective, screening is principally justified by prevention of blindness, and guidelines emphasize detection of vtDR (proliferative DR and/or centre‑involving diabetic macular oedema) [2]. Prior EyeArt evaluations have reported performance specifically for vtDR, often with sensitivity similar to that for any DR but with improved specificity when thresholds are tuned to this endpoint [3, 4]. In the present study, the near‑perfect sensitivity for any DR suggests that a modest relaxation of sensitivity in favor of higher specificity might be acceptable if the endpoint were vtDR, particularly in settings where referral capacity is constrained. Reporting EyeArt’s binocular sensitivity and specificity for vtDR in this cohort, and modelling expected referral volumes under different operating points, would therefore be highly informative.

The authors show that OCT‑defined diabetic macular edema and increased central retinal thickness are strongly associated with DR, and that these associations are recapitulated in AI‑based models. Yet EyeArt currently analyses only color fundus photographs. Taken together, these findings support development of multimodal AI systems that integrate fundus imaging with OCT (and potentially OCT‑angiography) to directly target vtDR rather than extrapolating from surrogate fundus features alone. Such systems could triage vtDR at the point of care, while fundus‑only AI remains an efficient front‑end option for clinics without OCT access.

Finally, the inverse association between age and DR after adjustment for diabetes duration hints at heterogeneous risk trajectories, consistent with prior epidemiologic data [2]. Incorporating age of onset and cumulative glycemic exposure into AI‑driven risk stratification, alongside image‑based outputs, might allow personalized screening intervals beyond a simple binary AI read‑out.

Re‑analysis of the Guedes et al. cohort using vtDR as the primary endpoint, and exploration of multimodal and risk‑integrated AI workflows, may provide readers with clinically actionable insight into how high‑performing algorithms such as EyeArt can be optimally deployed in real‑world screening programs.

## Data Availability

No datasets were generated or analysed during the current study.

## References

[1] Guedes II, Saavedra Santana P, Cabrera López F, Ramos Macías Á, Ramos de Miguel Á, González Hernández A. Evaluation of the degree of agreement in the diagnosis of diabetic retinopathy between ophthalmologists and eyeart. Int J Retina Vitreous. 2025;11:125. 10.1186/s40942-025-00748-4. 41257814 10.1186/s40942-025-00748-4 PMC12628637

[2] Ting DSW, Cheung GCM, Wong TY. Diabetic retinopathy: global prevalence, major risk factors, screening practices and public health challenges. Clin Exp Ophthalmol. 2016;44(4):260–77. 26716602 10.1111/ceo.12696

[3] Bhaskaranand M, Ramachandra C, Bhat S, et al. The value of automated diabetic retinopathy screening with the eyeart system: a study of more than 100,000 consecutive encounters from people with diabetes. Diabetes Technol Ther. 2019;21(11):635–43. 31335200 10.1089/dia.2019.0164 PMC6812728

[4] Lim JI, Regillo CD, Sadda SVR, et al. Artificial intelligence detection of diabetic retinopathy: subgroup comparison of the eyeart system with ophthalmologists’ dilated examinations. Ophthalmol Sci. 2023;3(1):100167. 10.1016/j.xops.2022.100228 PMC9636573 36345378

